# Caffeine Supplementation Strategies Among Endurance Athletes

**DOI:** 10.3389/fspor.2022.821750

**Published:** 2022-04-06

**Authors:** Andreas Kreutzer, Austin J. Graybeal, Kamiah Moss, Robyn Braun-Trocchio, Meena Shah

**Affiliations:** ^1^Department of Kinesiology, Texas Christian University, Fort Worth, TX, United States; ^2^School of Kinesiology and Nutrition, College of Education and Human Sciences, University of Southern Mississippi, Hattiesburg, MS, United States

**Keywords:** endurance performance, running, cycling, triathlon, caffeine, sports nutrition

## Abstract

Caffeine is widely accepted as an endurance-performance enhancing supplement. Most scientific research studies use doses of 3–6 mg/kg of caffeine 60 min prior to exercise based on pharmacokinetics. It is not well understood whether endurance athletes employ similar supplementation strategies in practice. The purpose of this study was to investigate caffeine supplementation protocols among endurance athletes. A survey conducted on Qualtrics returned responses regarding caffeine supplementation from 254 endurance athletes (f = 134, m =120; age = 39.4 ± 13.9 y; pro = 11, current collegiate athlete = 37, recreational = 206; running = 98, triathlon = 83, cycling = 54, other = 19; training days per week = 5.4 ± 1.3). Most participants reported habitual caffeine consumption (85.0%; 41.2% multiple times daily). However, only 24.0% used caffeine supplements. A greater proportion of men (31.7%) used caffeine supplements compared with women (17.2%; *p* = 0.007). Caffeine use was also more prevalent among professional (45.5%) and recreational athletes (25.1%) than in collegiate athletes (9.4%). Type of sport (*p* = 0.641), household income (*p* = 0.263), education (*p* = 0.570) or working with a coach (*p* = 0.612) did not have an impact on caffeine supplementation prevalence. Of those reporting specific timing of caffeine supplementation, 49.1% and 34.9% reported consuming caffeine within 30 min of training and races respectively; 38.6 and 36.5% used caffeine 30–60 min before training and races. Recreational athletes reported consuming smaller amounts of caffeine before training (1.6 ± 1.0 mg/kg) and races (2.0 ± 1.2 mg/kg) compared with collegiate (TRG: 2.1 ± 1.2 mg/kg; RACE: 3.6 ± 0.2 mg/kg) and professional (TRG: 2.4 ± 1.1 mg/kg; RACE: 3.5 ± 0.6 mg/kg) athletes. Overall, participants reported minor to moderate perceived effectiveness of caffeine supplementation (2.31 ± 0.9 on a four-point Likert-type scale) with greatest effectiveness during longer sessions (2.8 ± 1.1). It appears that recreational athletes use lower caffeine amounts than what has been established as ergogenic in laboratory protocols; further, they consume caffeine closer to exercise compared with typical research protocols. Thus, better education of recreational athletes and additional research into alternative supplementation strategies are warranted.

## Introduction

Endurance sports, including running, cycling, and triathlon, have been popular among recreational athletes in the USA. Prior to the COVID-19 pandemic the number of race registrations in running and triathlon alone was estimated between 22 and 30 million annually (Run Signup, 2021). Additionally, USA Cycling (USAC) members amassed over 300,000 racer days in 2019 (Vandivort, 2019). A common strategy to improve endurance performance is the use of caffeine as an ergogenic aid; up to 90% of recreational and professional endurance athletes use caffeine supplements to improve their performance (Desbrow and Leveritt, 2006, 2007; Del Coso et al., 2011). It has been shown that runners spend ~$1,000/race on marathon preparation and participation a significant proportion of which comes from nutritional supplements that often contain caffeine (Malito, 2016).

The benefits of caffeine supplementation for endurance performance have been well-established (Southward et al., 2018; Guest et al., 2021). Most laboratory protocols employ standardized procedures for caffeine administration: participants typically ingest 3–6 mg of caffeine per kilogram (mg/kg) of body mass 1 h prior to an exercise bout, which yields performance benefits of ~2–3% in time trial tasks compared with placebo controls (Southward et al., 2018). Even larger improvements have been demonstrated in time to exhaustion (TTE) tasks; e.g., Smirmaul et al. (2017) found a 12% improvement in TTE following ingestion of 4 mg/kg caffeine. An umbrella review of published meta-analyses by Grgic et al. (2019) revealed moderate effect sizes of caffeine supplementation for aerobic tasks and muscle endurance. In a smaller number of studies, lower doses of caffeine (≤ 3 mg/kg) administered at different time points before and during exercise have also been shown to be effective (Cox et al., 2002; Hogervorst et al., 2008; Talanian and Spriet, 2016).

Caffeine appears to exerts its ergogenic effect by binding to adenosine A_1_/A_2_ receptors and inhibiting the fatigue-inducing effects of adenosine in the brain (Davis et al., 2003). Additionally, de Morree et al. (2014) demonstrated decreased motor-related cortical potential and Ratings of Perceived Exertion (RPE) following caffeine ingestion. This reduction in RPE seems to be an important mechanism in the improved exercise performance afforded by caffeine supplementation (Doherty and Smith, 2005).

While the ergogenic effects of caffeine are widely accepted and its use is widespread, it appears that there remains some confusion among elite and recreational athletes regarding optimal supplementation protocols. More specifically, Desbrow and Leveritt (2006) reported 89% of participants, who competed in the 2005 Ironman World Championships, planned to use caffeine prior to or during the race; however, planned intake varied widely from 0 to 24 mg/kg, while actual intake averaged 4.9 ± 3.7 mg/kg. While that study provides a glimpse into caffeine supplementation habits among triathletes during a single race, to our knowledge no studies have investigated typical caffeine supplementation protocols across training and races among endurance athletes of various performance levels in a variety of sports. It is unclear if endurance athletes replicate supplementation protocols that have been shown to be successful in laboratory research.

Therefore, the purpose of this study was an exploratory examination of caffeine supplementation strategies among endurance athletes. We investigated caffeine supplementation prevalence, supplement type, timing, and amount, and the perceived effects of caffeine during training and racing using an online survey. Further, we collected data on where endurance athletes were receiving information regarding caffeine supplementation. Due to the descriptive and exploratory nature of this study, we did not pose any formal hypotheses.

## Method

### Study Design

We used an electronic survey (Qualtrics, Provo, UT) to investigate caffeine consumption, supplementation strategies, and perceived effects of caffeine among endurance athletes from June 2020 to March 2021. We recruited participants using social media, email, and word of mouth. The TCU Institutional Review Board (IRB) approved the study (Protocol # 1920-227). All participants signed an electronic informed consent. Participants who completed the survey were entered in a drawing to win one of 15 $100 gift cards.

### Participants

Participants were recruited using digital flyers distributed *via* email and social media groups. A total of 385 endurance athletes signed the informed consent, of whom 263 completed the survey. Individuals were included in the study if they were at least 18 years old and self-identified as endurance athletes. Endurance athletes were defined as those individuals who train and compete in activities that involve prolonged rhythmic exercise which are mainly powered by oxidative phosphorylation (Booth et al., 2015). Participants self-reported whether they were professional, collegiate or former collegiate athletes. Those who did not self-identify as either professional or current collegiate athletes, were categorized as recreational athletes. Exclusion criteria were self-reported age of ≤ 18 years, participation in a non-endurance sport, and failure to complete certain demographic or caffeine-related sections of the survey. Three participants were removed because they were participating in non-endurance sports (Basketball: *n* = 2; Tennis: *n* = 1, Crossfit/Olympic Weightlifting: *n* = 1). Six participants were excluded based on failure to report biological sex. Thus, we included 254 athletes in the final analysis.

### Questionnaire

We developed the questionnaire based on a published survey regarding pre-workout supplement use and personal communication with the first author of that study (Jagim et al., 2019). We worded demographic questions in accordance with the United States Census survey questions. We solicited feedback regarding the content validity and usability of the survey from experts in the field and from local endurance athletes during a pilot period. The questionnaire was administered electronically on Qualtrics and consisted of 12 demographic questions, 25 questions regarding sports background, and 64 questions regarding caffeine consumption and supplementation, e.g., caffeine capsules, energy gels, or caffeinated sports drinks. Question types included multiple choice, multiple selection, open-ended, and Likert-type questions. Participants were able to skip items at their own discretion. Further, the questionnaire contained skip-/display logic to display follow-up questions based on previous responses. Questions regarding habitual caffeine consumption were limited to caffeinated beverages. In an initial question, participants were asked whether they consume these types of beverages; based on their answer, they were presented with follow-up questions regarding the types and amounts consumed. When reporting amounts, participants chose from prespecified ranges of daily consumption volumes (<8 oz; 8–12 oz; 12–16 oz; 16–20 oz; >20 oz). Questions regarding caffeine supplementation were nested under an initial query on whether participants use any supplements containing caffeine. Follow-up questions regarding types, amounts, timing, motivation, and perceived effectiveness were displayed based on skip/display logic based on previous answers. Caffeine supplementation amounts were provided by participants in an open-ended question. This question was only displayed to participants who indicated that they used a specific amount of caffeine in a supplement. The survey can be found at https://osf.io/9grc2/.

### Data Handling and Recoding

Where participants reported caffeine amounts as ranges, e.g., 50–100 mg, we used the midpoint, e.g., 75 mg, for all analyses. We used the United States Department of Agriculture (USDA) National Nutrient Database for Standard Reference (Haytowitz et al., 2019) to estimate caffeine content, where participants reported caffeine amounts by referencing a beverage, e.g., one cup of coffee contains 95 mg of caffeine. Where caffeine amounts were reported by supplement, we referenced product websites to find caffeine content. We recoded timing of caffeine intake prior to races and training session from 15-min interval options to 30-min interval options due to the small number of responses for some answer choices. Participants rated caffeine effectiveness on a five-point Likert-type scale (0 = no effect, 1 = neutral, 2 = minor effect, 3 = moderate effect, 4 = major effect) considering a range of different situations characterized by the intensity and duration of training sessions and races as well as situations involving participants' feelings, including “lack of energy,” “sluggishness,” “general fatigue,” “muscle fatigue,” and “muscle soreness.” We averaged perception scores for races, training, and feelings for statistical analysis.

### Statistical Analysis

Statistical analyses were performed in jamovi version 2.2.2. We calculated descriptive statistics for age, height, weight, and BMI as means ± SD. Further we, calculated percentages for race, athlete status, training volume, education, household income, and primary sport as a percent of the total number of participants as well as by sex. Percentages are reported in relation to the number of participants responding to a particular question. We performed Pearson Chi-Square (χ^2^) tests to elucidate potential differences in caffeine supplementation strategies based on sex, primary sport, education, household income, athlete status (professional, collegiate, recreational), coaching, and recent race success. Alpha level for all tests was set to 0.05. We limited statistical analysis by primary sport to only runners, cyclists and triathletes, because none of the other groups were large enough to include (*n* ≤ 8). Additionally, we included one individual, who participated in aquabike (swim and run combination) in the group of triathletes. We compared participant characteristics between runners, cyclists, and triathletes using a one-way analysis of variance (ANOVA) with Tukey-corrected *post hoc* test. We confirmed normality of the residuals for these analyses using visual inspection of Q-Q plots. If a χ^2^ test comparing > 2 groups produced a statistically significant result, we performed pairwise comparisons by reducing the larger contingency table into multiple 2 × 2 tables and performing χ^2^ tests with a Bonferroni corrected alpha level (Kim, 2017). We compared perceived caffeine effectiveness based on whether it was used before a race, a training session, or based on participants feelings using a Friedman's repeated measures ANOVA with Durbin-Conover pairwise comparisons. Additionally, we compared perceived caffeine effectiveness between groups (primary sport, athlete status) using a Kruskal-Wallis one-way ANOVA. We employed these non-parametric analyses due to the ordinal nature of the data and the difference in group sizes in the analysis based on athlete status.

## Results

### Participant Characteristics

The participant characteristics are presented in Table 1. Our sample was mostly Caucasian (90.6%) and non-Hispanic (87.4%) and contained 52.8% females. Almost half of our sample held a graduate degree (47.2%), and more than half reported an annual household income > $100,000 (54.3%). The participants had a mean age of 39.4 ± 13.9 years, height of 172.0 ± 10.6 cm, weight of 71.2 ± 16.0 kg, and body mass index (BMI) of 24.0 ± 4.2 kg/m^2^. Majority of the participants were recreational athletes (81.1%), and the three most common primary sports were cycling (21.3%), running (38.6%), and triathlon (32.7%). The participants trained on average 5.4 ± 1.3 days per week with a median training time of 1.75 h per day. There was a significant effect of primary sport on age, *F*_(2, 233)_ = 25.13, *p* < 0.001, η^2^ = 0.177 and BMI, *F*_(2, 233)_ = 6.86, *p* = 0.001, η^2^ = 0.056. Cyclists were significantly older, *M* = 49.7 ± 12.4 years, compared with runners, *M* = 36.6 ± 11.6 years, *t*_(233)_ = 6.34, *p* < 0.001, and triathletes, *M* = 36.0 ± 12.8, *t*_(233)_ = 6.42, *p* < 0.001. Further, runners had the lowest body mass, *M* = 65.1 ± 13.3 kg, compared with triathletes, *M* = 71.1 ± 15.3 kg, *t*_(233)_ = −2.69, *p* = 0.021, and cyclists, *M* = 81.8 ± 17.1 kg, *t*_(233)_ = −6.59, *p* < 0.001.

**Table 1:** Participant characteristics.

	**M** **±SD or** ***n*** **(%)**		
**Variable**	**All**	**Female**	**Male**
	**(*n* = 254)**	**(*n* = 134)**	**(*n* = 120)**
Age (y)	39.4 ± 13.9	36.1 ± 12.7	43.1 ± 14.3
Height (cm)	172.0 ± 10.6	164.0 ± 7.2	179.0 ± 7.9
Weight (m)	71.2 ± 16.0	60.9 ± 10.3	82.6 ± 13.4
BMI (kg/m^2^)	24.0 ± 4.2	22.6 ± 3.9	25.7 ± 4.0
Race			
American Indian/Alaskan native	2 (0.8%)	1 (0.7%)	1 (0.8%)
Asian	8 (3.2%)	5 (3.7%)	3 (2.5%)
Black/African American	4 (1.6%)	2 (1.5%)	2 (1.7%)
Multiracial	7 (2.8%)	4 (3.0%)	3 (2.5%)
Native hawaiian/Pacific Islander	1 (0.4%)	1 (0.7%)	0 (0.0%)
Prefer not to answer	2 (0.8%)	1 (0.7%)	1 (0.7%)
White	230 (90.6%)	120 (89.6%)	110 (91.7%)
Athlete status			
Professional	11 (4.3%)	4 (3.0%)	7 (5.8%)
Collegiate (current)	37 (14.6%)	26 (19.4%)	11 (9.2%)
Collegiate (former)	43 (16.9%)	21 (19.8%)	22 (21.2%)
Recreational	206 (81.1%)	104 (77.6%)	102 (85.0%)
Training volume (hours/week)			
<5	15 (5.9%)	10 (7.5%)	5 (4.2%)
5–10	100 (39.4%)	48 (35.8%)	52 (43.3%)
10–15	85 (33.5%)	48 (35.8%)	37 (30.8%)
15–20	37 (14.6%)	23 (17.2%)	14 (11.7%)
20–25	12 (4.7%)	4 (3.0%)	8 (6.7%)
> 25	5 (2.0%)	1 (0.7%)	4 (3.3%)
Education			
High school diploma	7 (2.8%)	4 (3.0%)	3 (2.5%)
Vocational training	4 (1.6%)	1 (0.7%)	3 (2.5%)
Some college (<4 years)	38 (15.0%)	19 (14.2%)	19 (15.8%)
Bachelor's degree	85 (33.5%)	45 (33.6%)	40 (33.3%)
Graduate degree	120 (47.2%)	65 (48.5%)	55 (45.8%)
Income			
< $20,000	12 (4.7%)	7 (5.2%)	5 (4.2%)
$20,000–$34,999	14 (5.5%)	11 (8.2%)	3 (2.5%)
$35,000–$49,999	9 (3.5%)	6 (4.5%)	3 (2.5%)
$50,000–$74,999	30 (11.8%)	13 (9.7%)	17 (14.2%)
$75,000–$99,999	22 (8.7%)	16 (11.9%)	6 (5.0%)
> $100,000	138 (54.3%)	63 (47.0%)	75 (62.5%)
Prefer not to answer	29 (11.4%)	18 (13.4%)	11 (9.2%)
Primary sport			
Cycling	54 (21.3%)	11 (8.2%)	43 (35.8%)
Para-cycling	1 (0.4%)	1 (0.7%)	0 (0.0%)
Rowing	7 (2.8%)	3 (2.2%)	4 (3.3%)
Running	98 (38.6%)	68 (50.7%)	30 (25%)
Snowshoeing	1 (0.4%)	1 (0.7%)	0 (0.0%)
Swimming	7 (2.8%)	4 (3.0%)	3 (2.5%)
Triathlon	83 (32.7%)	45 (33.6%)	38 (31.7%)
Wheelchair racing	2 (0.8%)	1 (0.7%)	1 (0.8%)

### Habitual Caffeine Consumption

Most participants reported habitual consumption of caffeinated beverages (*n* = 216; 85.0%) with many of them consuming these beverages multiple times daily (*n* = 89; 41.2%) or once daily (*n* = 79; 36.6%). Additionally, some participants reported consuming caffeinated beverages occasionally (*n* = 8; 3.7%), on most days each week (*n* = 23; 10.7%) or on some days each week (*n* = 17; 7.9%). Preferred beverages among were brewed coffee (*n* = 166 out of 216; 76.9%), caffeinated teas (*n* = 68; 31.5%), caffeinated soft drinks (*n* = 53; 24.5%), plain espresso (*n* = 27; 12.5%), and energy drinks (*n* = 26; 12.0%). The most frequently reported daily quantity of caffeinated beverages was 8–12 fluid ounces (oz) (*n* = 65; 30.2%), followed by 12–16 oz (*n* = 53; 24.7%), 16–20 oz (*n* = 39; 18.1%), and more than 20 oz (*n* = 36; 16.7%. Twenty-two participants (10.2%) reported consuming less than 8 oz of caffeinated beverages per day. A significantly larger proportion of men (*n* = 110; 91.7%) reported habitual caffeine consumption when compared with women (*n* = 106, 79.1%), χ^2^ (1) = 7.850, *p* = 0.005, *V* = 0.18.

### Caffeine Supplementation Prevalence and Motivation

Sixty-one participants (24.0%) reported using caffeine supplements. Among these participants, 60.7% (*n* =37) reported using sports/energy gels, 23.0% (*n* = 14) reported using sports/energy drinks and/or sports/energy gummies, and 16.4% (*n* = 10) reported using pre-workout powders. Eight participants (13.1%) used caffeine tablets, six (9.8%) used other powders containing caffeine, and four (6.6%) used caffeinated sports/energy chewing gum. Reasons for caffeine supplementation included the belief that there are performance and cognitive/mental benefits (*n* = 31; 50.8%), performance benefits only (*n* = 15; 24.6%), and cognitive/mental benefits only (*n* = 1; 1.6%). Eight participants (13.1%) reported using caffeine supplements only when they feel tired.

When asked where they got the recommendation to use caffeine supplements, 36.1% (*n* = 22) of participants indicated that a fellow athlete recommended supplementation. Fifteen participants (24.6%) reported getting the recommendation from a website and 10 participants (16.4%) reported using a scientific research document as their source for recommendations. While nine participants (14.8%) received recommendations to supplement with caffeine from a coach, only two participants (3.3%) reported getting recommendations from a nutritionist or dietitian.

Many of those not taking caffeine supplements reported that they do not use these supplements because they do not like the way they make them feel (*n* = 67; 34.7%). Other reasons for not supplementing with caffeine included that it does not provide performance benefits (*n* = 38, 19.7%) or cognitive/mental benefits (*n* = 28; 14.5%). Medical reasons (*n* = 17; 8.8%) and high expenses (*n* = 12; 6.2%) were other reasons. Approximately 30% of participants (*n* = 57) wrote in their own reasons; common themes among these included relying on coffee intake, concerns regarding caffeine's effect on hydration, and increased heart rate.

**Figure 1:**
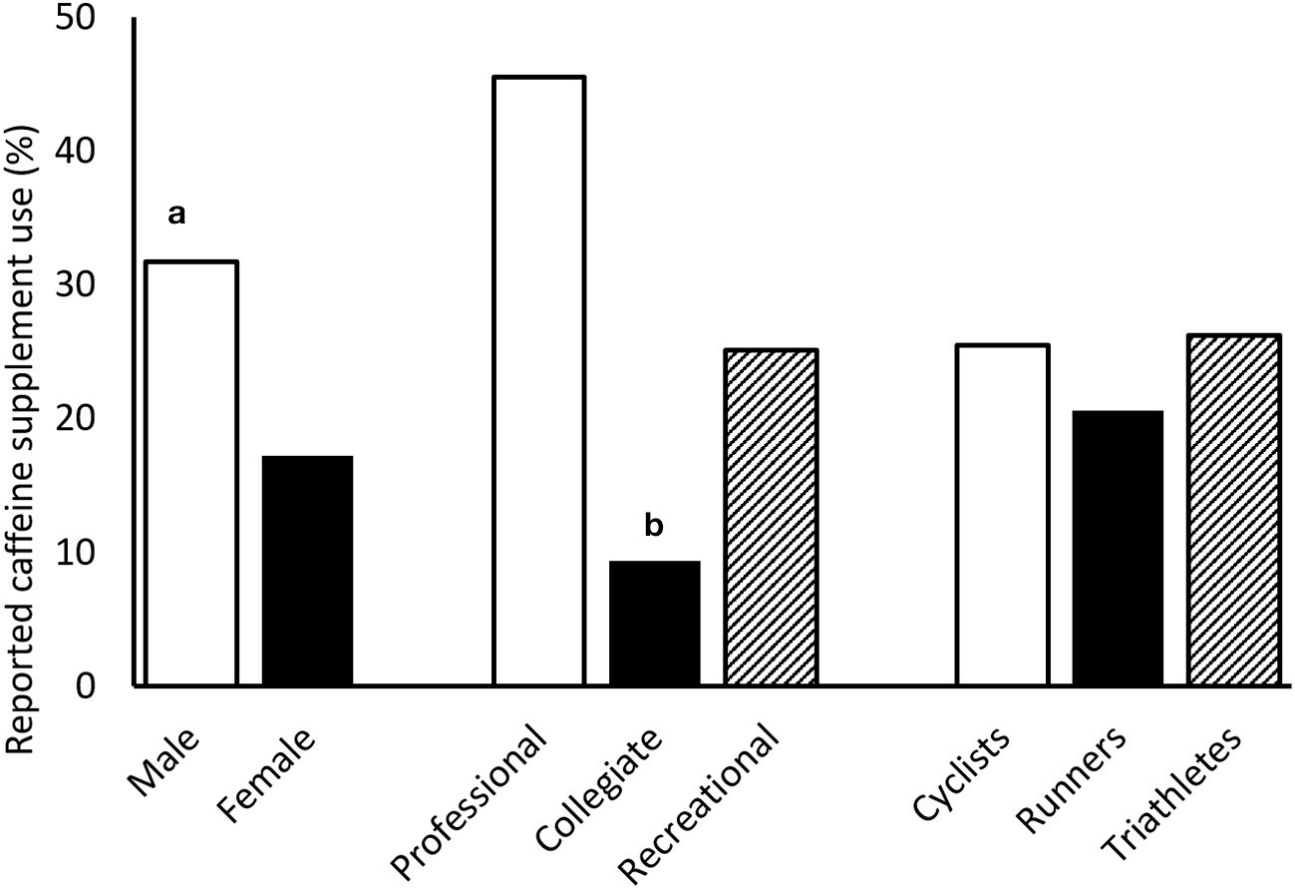
Reported caffeine supplementation use by sex, athlete status, and primary sport. **(A)** Significantly greater than Female; **(B)** Significantly lower than Professional and Recreational.

Caffeine supplementation was more prevalent among men (*n* = 38; 31.7%) when compared with women (*n* = 23; 17.2%), χ^2^ (1) = 7.30, *p* = 0.007, *V* = 0.17. Additionally, there were significant differences in caffeine supplementation frequency by athlete status, χ^2^ (2) = 6.67, *p* = 0.036, *V* = 0.16. Collegiate athletes reported a lower prevalence of caffeine supplementation (*n* = 3; 9.4%) when compared with recreational athletes (*n* = 53; 25.1%), χ^2^ (1) = 3.88, *p* = 0.049, *V* = 0.13 and professional athletes (*n* = 5; 45.5%), χ^2^ (1) = 7.04, *p* = 0.008, *V* = 0.41. There were no differences in caffeine supplementation frequency when comparing runners, cyclists, and triathletes, χ^2^ (2) = 0.890, *p* = 0.641, *V* = 0.06. Similarly, we found no differences when comparing caffeine supplementation rates based on education, income, or race. Interestingly, caffeine supplementation was higher among those who finished in the Top 3 of their age and sex division in at least one race during the previous year (*n* = 33; 30.3%), compared with those who did not achieve a Top 3 finish (*n* = 28; 19.3%), χ^2^ (1) = 4.10, *p* = 0.043, *V* = 0.13. Caffeine supplementation prevalence is presented in Figure 1.

### Caffeine Use Timing

Similar proportions of athletes reported using any type of caffeine, including specific supplements and caffeinated beverages, for training sessions (*n* = 71; 28.1%) and races (*n* = 82, 32.4%). Among those reporting caffeine use for training, 28 (40%) used caffeine only before some training sessions and 14 (20%) used caffeine only before all their sessions. An additional 14 participants (20%) reported using caffeine before and during some training sessions. Finally, 12.9% (*n* = 9) reported using caffeine only during some training sessions. Athletes reported that they determined whether to use caffeine supplements before training sessions based on the planned duration and intensity (*n* = 23; 46.0%), based on planned duration only (*n* = 14; 28.0%), or based on planned intensity only (*n* =10; 20.0%).

**Figure 2:**
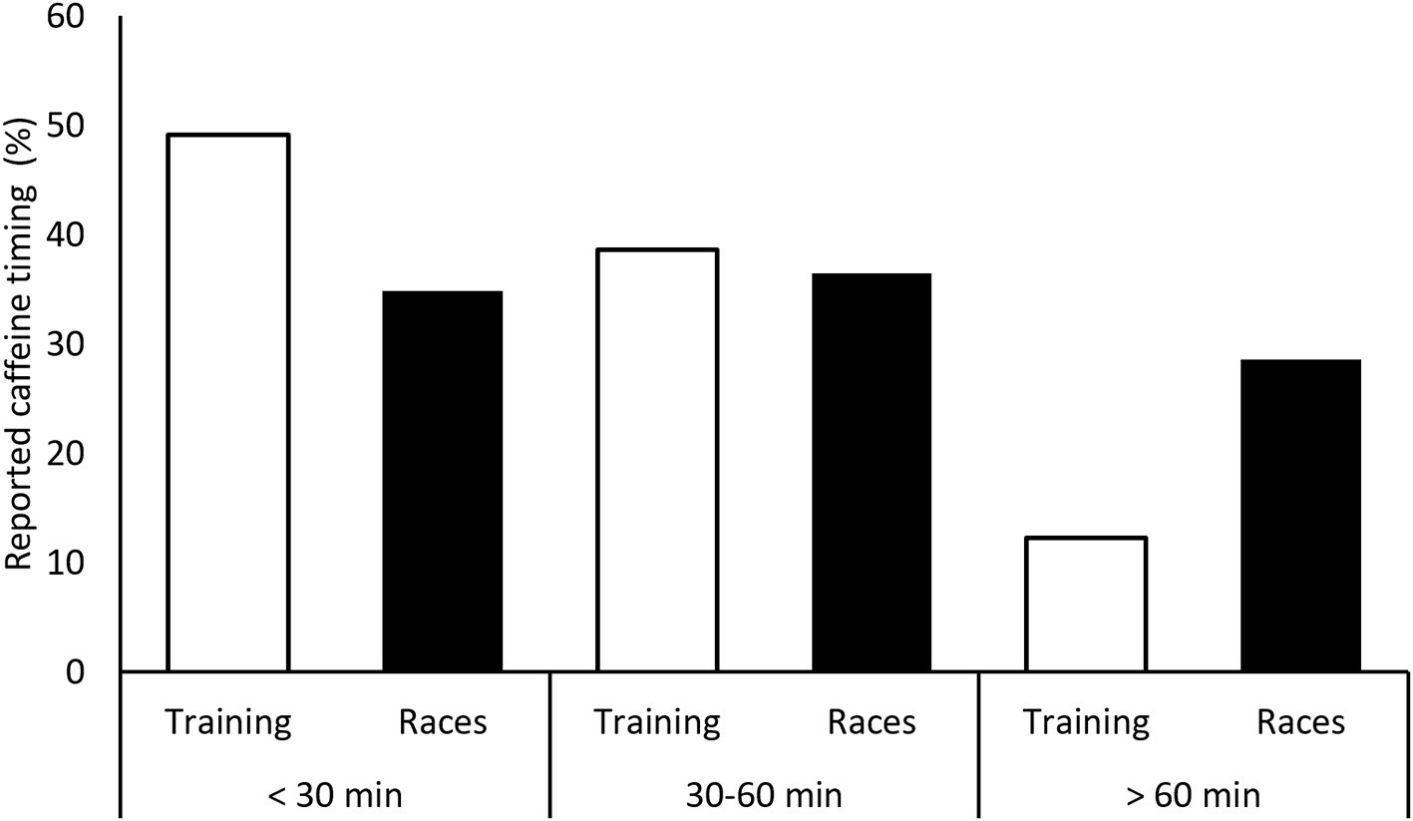
Reported caffeine intake timing prior to training and races.

When using caffeine for races, many athletes reported ingesting it only before all races (*n* = 33; 40.7%). Similar proportions reported using caffeine before and during all races (*n* = 12; 14.8%), before and during some races (*n* = 13; 16.1%), and only before some races (*n* = 11; 13.6%). Among those reporting specific timing of caffeine intake before training, 49.1% (*n* = 28) ingested caffeine < 30 min before sessions, 38.6% (*n* = 22) ingested caffeine 30–60 min before sessions, and 12.3% (*n* = 7) ingested caffeine >60 min before training. Results regarding caffeine intake timing before races were similar: 22 participants (34.9%) reported caffeine use < 30 min before races, 23 participants (36.5%) used caffeine 30–60 min before races, and 18 participants (28.6%) supplemented > 60 min before races. Surprisingly, among those who reported caffeine intake only before races, 40% (*n* = 16) took caffeine > 60 min prior to race start, whereas only 12.5% (*n* = 3) of participants reporting caffeine intake before and during races supplemented > 60 min prior to the start. Figure 2 shows caffeine supplementation timing prior to races and training sessions. Out of those taking caffeine during races, 72.0% (*n* =18) reported taking multiple doses of caffeine; most of them (54.2%; *n* = 13) reported taking caffeine every 60–90 min.

### Caffeine Supplementation Amount

Among those reporting caffeine supplementation for training and racing, 27.6% (*n* = 16) used a specific amount of caffeine, whereas 72.4% reported not using a specific amount. Most of the participants using a prespecified amount reported that personal experimentation (*n* = 11; 68.8%) led them to take a pre-specified amount. Other reasons for taking a pre-specified amount included advice from a coach (*n* = 3; 18.8%), advice from a friend or relative (*n* = 2; 12.5%), and advice from a nutritionist or dietitian (*n* = 2; 12.5%). Half of the participants who reported using a pre-specified amount of caffeine (*n* = 8) based the amount on a typical serving of their preferred supplement. Five participants (31.3%) based the amount on the total amount of caffeine they wanted to consume at the time. One participant specified that they based the amount on National Collegiate Athletic Association (NCAA) regulations, and another determined it based on a genetic test. The final participant reported taking in 100 mg per hour of training session or race. Interestingly, no participants used a relative measure (mg/kg) for dosing.

**Figure 3:**
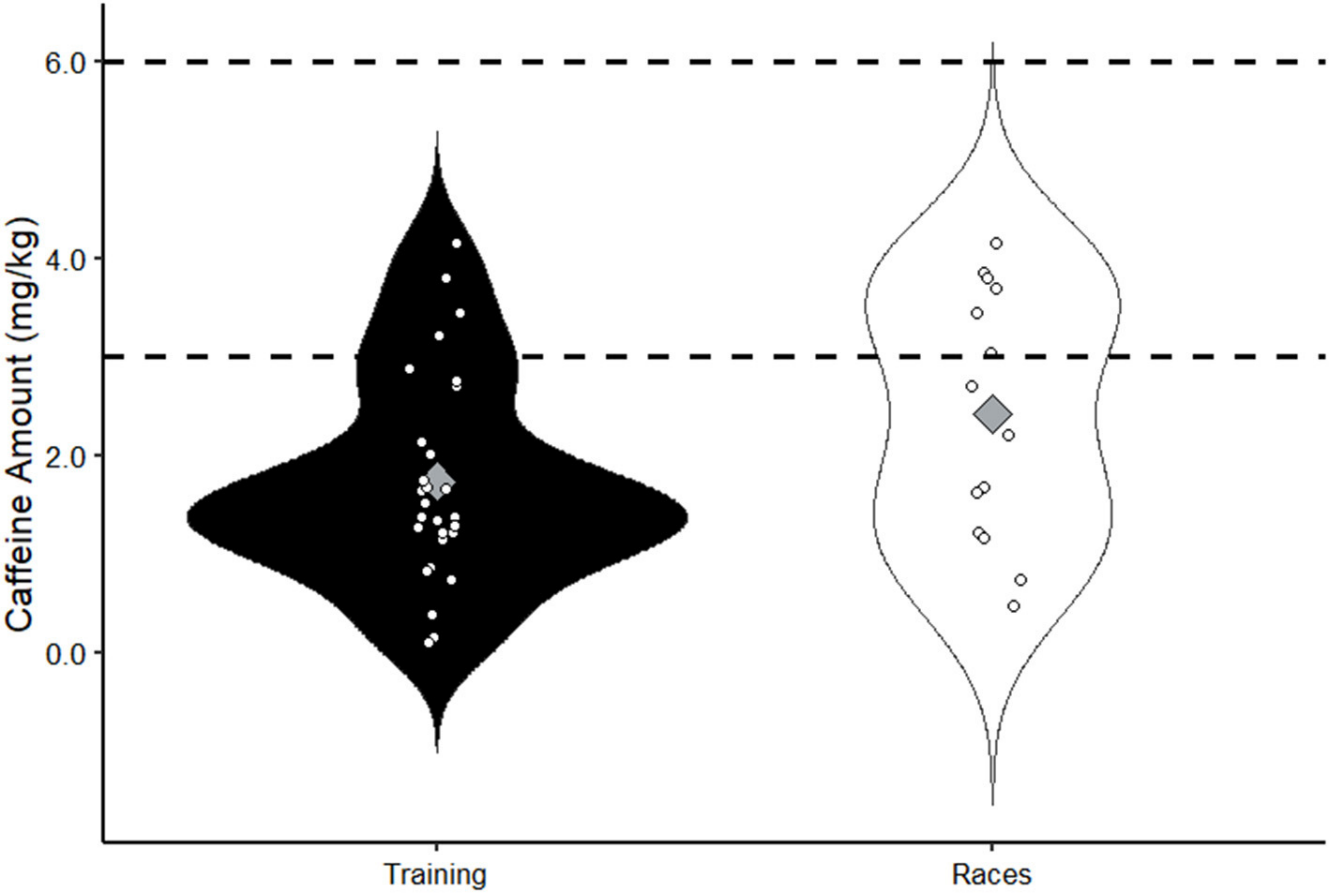
Reported caffeine amount ingested prior to training and races; white dots represent individual data points; gray diamonds represent means; dashed lines show typical caffeine amounts administered 60 min before exercise in laboratory studies (3–6 mg/kg).

On average, athletes used 124.0 ± 76.7 mg (1.7 ± 1.1 mg/kg) when supplementing only before training sessions (*n* = 28) and 168.0 ± 90.7 mg (2.4 ± 1.3 mg/kg) only before races (*n* = 13). Participants used less caffeine during training only [*n* = 6; 45.9 ± 37.8 mg (0.6 ± 0.5 mg/kg)] and when employing combined strategies [before and during training; *n* = 10; 94.5 ± 79.6 mg (1.2 ± 0.9 mg/kg)]. There was no difference in caffeine supplementation amounts when comparing men and women, nor when comparing cyclists, triathletes, and runners. Collegiate and professional athletes appeared to report higher caffeine intakes before training (COL: 2.1 ± 1.2 mg/kg; PRO: 2.4 ± 1.1 mg/kg) and racing (COL: 3.6 ± 0.2 mg/kg; PRO: 3.5 ± 0.6 mg/kg) compared with recreational athletes (TRG: 1.6 ± 1.0 mg/kg; RACE: 2.0 ± 1.3 mg/kg). We did not perform statistical analyses on these data due to small and uneven group sizes. Caffeine supplementation amounts before races and training sessions are shown in Figure 3.

### Perceived Effectiveness

There was a significant effect of supplementation situation (training vs. race vs. based on feelings) on the perceived effectiveness of caffeine, χ^2^ (2) = 20.700, *p* < 0.001. Participants reported greater caffeine effectiveness in races, *M* = 2.6 ± 1.1 when compared with training, *M* = 2.2 ± 0.9, *DC* = 5.00, *p* <0.001, and situations where they used caffeine based on feeling, *M* = 2.4 ± 1.0, *DC* = 2.14, *p* = 0.034. Additionally, they reported greater effectiveness when using caffeine based on a feeling compared with taking it during training, *DC* = 2.86, *p* = 0.005.

There was a significant effect of session length on the perceived effectiveness of caffeine supplementation, χ^2^ (2) = 41.600, *p* <0.001. Participants perceived caffeine to be more effective during longer races and training sessions, *M* = 2.8 ± 1.1) when compared with moderate-duration sessions, *M* = 2.3 ± 1.0, *DC* = 4.41, *p* <0.001, and short sessions, *M* = 1.9 ± 1.0, *DC* = 8.15, *p* <0.001. Perceived effectiveness was also significantly greater during moderate-duration training and races compared with short duration sessions, *DC* = 3.74, *p* <0.001.

There was no difference in the perceived effectiveness of caffeine when comparing women and men, *t*_(51)_ = 0.280, *p* = 0.781, *d* = 0.08. Similarly, perceived effectiveness was similar between cyclists, runners, and triathletes, χ^2^ (2) = 1.730, *p* = 0.422, ε^2^ = 0.04, as well as between professional, collegiate, and recreational athletes, χ^2^ (2) = 4.090, *p* = 0.130, ε^2^ = 0.08.

### Adverse Effects

Among those supplementing with caffeine, 12 participants (20.7%) reported having experienced adverse effects of caffeine ingestion. Ten of them (83.3%) experienced those effects two to four times, and two of them (16.7%) reported experiencing them more than six times. The most common adverse effects were difficulty sleeping (*n* = 7; 58.3%), jitteriness (*n* = 7; 58.3%), anxiety (*n* = 5; 41.7%), nervousness (*n* = 5; 41.7%), digestive issues (*n* = 5; 41.7%), and rapid heart rate (*n* = 4; 33.3%).

## Discussion

The present study aimed to investigate caffeine supplementation strategies employed by endurance athletes from a wide range of sports. While the ergogenic effect of caffeine for endurance performance is well established (Southward et al., 2018), most laboratory protocols use moderate to high doses of caffeine (36 mg/kg) ~60 min prior to exercise. Our main finding was that endurance athletes, especially recreational athletes, ingest lower amounts of caffeine before training and racing than those typically used in the laboratory setting. Collegiate and professional athletes used amounts at the lower end of the established range. Similarly, most participants in our sample ingested caffeine closer to exercise than the 60 min used in scientific research.

### Caffeine Dose

In a recent review, Grgic (2021) have suggested a minimal effective dose of 1.5 mg/kg for improving resistance exercise performance, which is similar to what participants in the present study reported using prior to training sessions. A minimal effective dose for endurance performance benefits has not been established, and studies on low-dose caffeine supplementation have shown mixed effects. Kovacs and Stegen (1998) demonstrated that 2.1, 3.2, and 4.5 mg/kg of caffeine all improved cycling performance in a ~1-h time trial (TT); however, performance improved more with higher doses. In a study of even lower doses, Jenkins et al. (2008) reported no performance benefits of 1 mg/kg of caffeine on work completed during a 15-min maximal cycling bout. However, they did report performance improvements in the same task with 2 mg/kg (4% improvement) and 3 mg/kg (3% improvement). Wiles et al. (2006) sought to investigate a more applied scenario by asking participants to ingest coffee containing 150–200 mg of caffeine (an amount similar to 1.5–2 eight oz cups of brewed coffee) prior to three different exercise protocols. The study did not report caffeine amounts in relative units and did not provide participants' body mass. However, the participants are described as male middle distance runners, so an estimated body mass of ~65 kg seems appropriate, meaning they ingested ~2.3–3.1 mg/kg of caffeine. Supplementation in that study improved 1,500 m running time, increased speed during an end spurt, and increased VO_2_ during a constant-speed high-intensity 1,500 m run compared with decaffeinated coffee. Desbrow et al. (2009) showed no ergogenic effects of 1.5 or 3 mg/kg of caffeine on performance in a 7 kJ/kg body weight TT, which was performed following 120 min of steady-state cycling at 70% of peak oxygen consumption (VO_2_peak).

Based on these results, it appears that while the amount ingested by endurance athletes in the present sample could be enough to confer performance benefits, higher doses might be more beneficial. As discussed above, none of our participants reported using dosing strategies relative to body mass. This could indicate that caffeine supplementation based on absolute amounts contained in commercially available multi-ingredient products could provide enough of a stimulus to be ergogenic, but that greater amounts of caffeine tailored to an individual's body weight could prove more beneficial. In our sample, participants reported greater caffeine effectiveness during races when compared with training; they also reported higher caffeine intake during races compared with training, suggesting that these greater amounts might be perceived more beneficial than the lower amounts used in training. Thus, more education of recreational athletes and their coaches is needed to optimize and individualize these strategies.

### Caffeine Timing

Most research investigating the ergogenic effects of caffeine has administered caffeine 60 min prior to exercise, since caffeine concentration in the blood peaks around this time (Graham and Spriet, 1995), especially in a fasted state (Skinner et al., 2013). In the fasted state, serum caffeine concentration begins to decrease after 60 min and continues to decrease through 240 min (Skinner et al., 2013). In the fed state, caffeine peaks later, at ~180 min following ingestion, but exhibits a lower peak compared with the fasted state. Most of the athletes in the present sample reported taking caffeine closer to exercise. This might in fact be the better strategy, especially in longer training sessions or races, where the effects of caffeine would be most important later in the race. Participants' perception of effectiveness reflected this notion, as they rated the effects of caffeine higher the longer the duration of training or races was. While no studies have shown that the performance is optimized at peak caffeine concentrations, recent work by Harty et al. (2020) suggests that caffeine ingestion 60 mins prior to performing explosive/strength tasks was more beneficial than ingestion 30 or 120 mins prior. Yet, this might differ for endurance performance, due to the prolonged nature of endurance tasks.

Several studies have investigated the effect of caffeine administration during exercise in an attempt to elucidate the effect of increasing caffeine concentration in the blood later in the performance bout (Kovacs and Stegen, 1998; Cox et al., 2002; Hogervorst et al., 2008; Talanian and Spriet, 2016). Cox et al. (2002) investigated the effects of 6 mg/kg caffeine administered 1 h prior to 120 min of submaximal cycling, 6 × 1 mg/kg administered every 20 min, 2 × 5 ml/kg Coca-Cola (0.7 mg/kg total) administered at 100 min and 120 min on performance in a subsequent 7 kJ/kg TT. They showed similar performance improvements (~3%) in all conditions compared with placebo. In a follow-up study presented in the same manuscript, Cox et al. administered the Coca-Cola at 80 and 100 based on participant feedback from Study 1 and allowed additional consumption of 5 ml/kg Coca-Cola during the TT. This second study showed similar performance improvements compared to Study 1. Talanian and Spriet (2016) asked participants to complete a 120-min cycling challenge, which consisted of cycling at 60% of VO_2_peak interspersed with five 120-second high-intensity intervals, before completing a 6 kJ/kg TT. Participants received 1.5 or 2.9 mg/kg of caffeine 80-min into the cycling challenge. TT performance improved in both caffeine conditions when compared with a placebo condition; this improvement was greater with the higher dose of caffeine.

Some of our participants reported taking caffeine before and during (20%) or only during (13%) training sessions. In these situations, participants reported taking 1.2 ± 0.9 and 0.6 ± 0.5 mg/kg, respectively. While these doses are lower than those reported to achieve the biggest performance improvements in laboratory studies, they are consistent with the lower doses also reported to confer some benefits. In the research setting, caffeine has been administered every 20 min or close to the end of a submaximal exercise bout prior to a TT. However, based on our study, it appears that endurance athletes take caffeine less frequently (54.2% took it every 60–90 min). They often take more than one dose per exercise bout (72%) and base the amount on a typical serving of their preferred sports supplement.

### Caffeine Supplementation Prevalence

To our knowledge, this is the first study to investigate caffeine supplementation prevalence among a range of endurance athletes. While a study by Desbrow and Leveritt (2006) suggested a high prevalence of targeted caffeine use among triathletes (89% of participants, who competed in the 2005 Ironman World Championship, planned to use caffeine during the race), the present study paints a different picture. Only 28.1% of our participants reported using caffeine for training and 32.4% reported using it for races. This stark difference could in part be explained by the fact that the Ironman World Championship represents the pinnacle of the sport of triathlon; therefore, athletes might be more inclined to use any performance enhancing strategies available to them. Additionally, race times in the study by Desbrow and Leveritt (2007) ranged from ~8 to 16 h. The long duration of this event might have led participants to consume caffeine to delay fatigue. In the present study, we asked for general supplementation patterns during all training and racing. Interestingly, we also found higher caffeine use prevalence in those finishing in the Top-3 of their division in the previous year. To qualify for the Ironman World Championship, athletes must finish in a qualifying slot in a full-distance triathlon; while the number of qualifying slots differs from race to race based on the number of competitors racing in each age group, qualifying often requires athletes to finish in the Top 3 in their age group. Thus the sample in Desbrow and Leveritt (2006) might have had an overrepresentation of those who supplement with caffeine. It stands to reason that those who pursue success at the highest level in their division are more likely to supplement with caffeine compared with those who do not. In our sample, those who had placed in the Top 3 overall (*n* = 23; 44.2% used caffeine) or in their age group (*n* = 45; 41.3%) in the past year were more likely to use caffeine before races than those who didn't (*n* = 58–37; 25.7–29%).

In a survey study similar to ours, Chester and Wojek (2008) reported that caffeine intake with the goal of performance improvement was greater among cyclists (59.9%) compared with track and field athletes (32.6%). We found no difference in caffeine supplementation prevalence when comparing cyclists, runners, and triathletes. The sample of track and field athletes in Chester and Wojek (2008) comprised 81% endurance athletes and 19% power athletes, thus making a direct comparison to our results difficult. However, the overall prevalence of caffeine supplementation was closer to the one reported here.

In the present study, caffeine supplementation was more prevalent in men compared with women. Aguilar-Navarro et al. (2020) reported that dietary supplement use among Spanish elite athletes was greater in men (65.3%) compared with women (56.6%). Interestingly, they reported greater caffeine supplement use among women (~5%) compared with men (~2%). It is possible that in our sample the greater prevalence for supplement use in men extended to caffeine as well. While limited research investigating the ergogenic effects of caffeine in women exists, it appears that these effects might be smaller and less consistent than in men (Mielgo-Ayuso et al., 2019; Harty et al., 2020). Thus, women might be less likely to use caffeine for performance improvement because they are unable to find research showing its efficacy for them. Additionally, the study by Aguilar-Navarro et al. (2020) was completed in elite athletes, who might have worked with dietitians and nutritionists and who had to follow anti-doping guidelines for the use of nutritional supplements.

Collegiate athletes in our study were significantly less likely to report the use of caffeine supplements compared with professional and recreational athletes. This might be in part because collegiate athletes have to comply with NCAA guidelines, which set an acceptable urine caffeine concentration at ≤ 15 μg/mL (~500 mg of caffeine); concentrations above this threshold would constitute a positive test for a banned substance. Thus, it stands to reason that collegiate athletes might be hesitant to report caffeine supplementation in a survey, even if they take caffeine in amounts that are allowed according to NCAA guidelines (Fralick and Braun-Trocchio, 2019). Interestingly, in a study by Froiland et al. (2004), 72.9% of collegiate athletes reported consuming energy drinks, which typically contain caffeine, but only 11.1% reported using caffeine supplements. Thus, the prevalence reported in the present study might be an underrepresentation of actual caffeine supplement use among collegiate athletes.

### Habitual Caffeine Consumption

Habitual caffeine consumption was reported by 85% of participants in the present study, which is similar to the results of a study by Frary et al. (2005), who surveyed 18,081 participants in the United States and reported 87% of participants consuming caffeine. The types of caffeinated beverages preferred was also similar between our study and that of Frary et al. (2005) with over 70% of participants reporting the consumption of coffee-based drinks and 23% (Frary et al., 2005) and 30% (present study) of participants consuming caffeinated teas. Caffeinated soft drink consumption was slightly higher in the study by Frary et al. (2005) (30.6%) compared with our results (24.5%). This could be because our sample was highly educated and highly active; thus, our participants may have been particularly health conscious and aware of the negative health impacts of soft drink consumption in general.

Several studies have investigated the effect of habitual caffeine consumption on the ergogenic effect of caffeine supplementation (Dodd et al., 1991; Bell and McLellan, 2002; Beaumont et al., 2017; Gonçalves et al., 2017; Lara et al., 2019; de Salles Painelli et al., 2021; Grgic and Mikulic, 2021). While some studies have reported greater benefits of caffeine supplementation when not habitually consuming caffeine (Bell and McLellan, 2002; Beaumont et al., 2017; Lara et al., 2019), they still have shown improved performance compared to placebo even with habitual caffeine intake. Other studies have shown no effect of habitual consumption on the potency of caffeine supplementation for performance enhancement (Dodd et al., 1991; Gonçalves et al., 2017; de Salles Painelli et al., 2021; Grgic and Mikulic, 2021). Thus, while our study shows that endurance athletes exhibit a habitual caffeine consumption prevalence similar to the general public, this should not be a concern regarding the efficacy of acute caffeine supplementation before training and races. In fact, some of our subjects indicated that they incorporate their daily coffee or tea consumption into their nutritional strategies before and during training.

### Mode of Delivery

Our participants reported using energy gels as their source of caffeine during training and races. These caffeinated energy gels typically contain carbohydrate, electrolytes, and caffeine. To our knowledge, no studies have investigated the pharmacokinetics of caffeine co-ingested with carbohydrate in energy gels. Skinner et al. (2013) showed that caffeine ingested immediately following a high-carbohydrate meal reached peak serum conditions ~ 2 h later than caffeine ingested in a fasted state. Additionally, peak serum caffeine concentrations for both 6 and 9 mg/kg doses were lower following co-ingestion with carbohydrate compared to the fasted state. Skinner et al. (2013) also showed that serum paraxanthine concentrations, the main metabolite of caffeine in humans, which is assumed to play a role in caffeine's ergogenic effects by acting as an adenosine receptor antagonist (Müller and Jacobson, 2011), were blunted in the fed state compared to the fasted state. Thus, co-ingestion of carbohydrate and caffeine could potentially reduce caffeine's ergogenic effect, especially since many energy gels contain relatively low amounts (20–75 mg) of caffeine.

Yet, carbohydrate ingestion on its own is an established strategy to improve endurance performance (Jeukendrup, 2004) and the effects of co-ingestion with caffeine appear to be unclear. Yeo et al. (2005) reported that exogenous carbohydrate oxidation was enhanced by co-ingestion of 5 mg/kg of caffeine during the final 30 min of a 120-min cycling bout at 64 ± 3% of VO_2_max. In a follow-up study in the same laboratory, Hulston and Jeukendrup (2008) were unable to replicate this difference in exogenous carbohydrate oxidation, but did demonstrate that performance in a subsequent TT was augmented with co-ingestion of 5.3 mg/kg of caffeine and a 6.4% glucose solution compared with carbohydrate alone and placebo. However, Barzegar et al. (2021) recently showed that the ingestion of four doses of 0.6 g/kg of carbohydrate alone and along with 6 mg/kg caffeine over a 12-h period following exhaustive exercise similarly improved average 500-m paddling times on the next day in highly trained participants. Performance was not augmented by the co-ingestion of caffeine with carbohydrate over and above the effects of carbohydrate alone. It is important to note that the latter study aimed to investigate the effects of caffeine-carbohydrate co-ingestion on muscle glycogen resynthesis, and thus caffeine and carbohydrate ingestion ceased 12-hs prior to the performance bout. Additionally, the five 500-m bouts employed in the study by Barzegar et al. (2021) were significantly shorter (90–100 sec) compared with the cycling TT (~45 min) in the investigation by Hulston and Jeukendrup (2008). Thus, it appears that caffeine-carbohydrate co-ingestion is more effective when performed acutely before or during endurance exercise, similar to what our participants reported.

Few studies have investigated the effect of caffeinated energy gel consumption on exercise performance. Cooper et al. (2014) showed improved fatigue index and lower RPE in the third and fourth block of a four-block intermittent sprint test following caffeinate gel (100 mg caffeine, 25 g carbohydrate) ingestion compared with placebo and carbohydrate alone. Similarly, Scott et al. (2015) reported improved 2,000 m rowing performance after using a caffeinated gel (100 mg caffeine, 21.6 g carbohydrate) when compared with a carbohydrate gel only. In a study of resistance trained men, Venier et al. (2019), showed improvements in jump height, muscle strength, and muscle power following ingestion of an energy gel containing 300 mg caffeine and 88 g carbohydrate compared with carbohydrate-only gel. While these studies show that energy gels can be ergogenic, no studies have compared the use of energy gels to similar doses of caffeine ingested in isolation.

Another caffeine source that has recently gained popularity are caffeinated chewing gums. Some of our participants reported using these gums as their mode of delivery for caffeine. A study by Kamimori et al. (2002) showed that caffeine is absorbed faster from chewing gum compared with caffeine capsules, likely because absorption is facilitated through the buccal mucosa. Most studies comparing caffeinated chewing gum with placebo conditions and/or multi-ingredient caffeine supplements have shown performance improvements (Ryan et al., 2013; Lane et al., 2014; Paton et al., 2015). However, it appears that no studies have compared the effect caffeinated chewing gum with pure caffeine capsules or powder on endurance performance. Lane et al. (2014) showed that plasma caffeine concentrations were similar between caffeinated chewing gum and beetroot juice with caffeine. Both caffeinated supplements also showed similar performance improvements compared to placebo and beetroot juice alone. Ryan et al. (2013) reported that caffeinated chewing gum (300 mg caffeine) was effective in improving 7 kJ/kg cycling TT performance compared with placebo only when the gum was administered 5 min prior to 15-min steady-state cycling, which preceded the TT. When administered 60 min or 120 min prior to exercise, performance was not improved by the caffeinated gum. Thus, while chewing gum might be an effective option for caffeine supplementation, it is important to consider timing of ingestion and the length of the exercise bout.

### Limitations

One of the limitations of the present study is its reliance on self-report and, thus, on the assumption of truthfulness in the responses of participants. Additionally, this survey was conducted during the height of the COVID-19 pandemic, when typical training and racing behaviors were disrupted by lockdowns and event cancellations. Thus, the data reported by some of the participants might not accurately reflect their typical behaviors. Another shortcoming of the survey is that it did not specifically ask participants about their motivation to use caffeine during training. While the use during races is tied to improvements in performance, the benefits during training, e.g., increase in tolerable duration or intensity, are less clear. Future research should investigate these motivations. Additionally, the duration to complete the survey could have led to respondent fatigue, which might be reflected in the limited number of participants who completed questions about the exact amount and timing of caffeine supplementation. Future studies should limit the number of questions and attempt to focus on a narrower concept, e.g., a strong focus on caffeine timing and dosing in specific situations. Finally, the numbers of professional, collegiate, and recreational athletes participating in the study were unequal limiting generalizability. Moreover, the unequal groups may have affected the findings on perceived effectiveness of caffeine by athlete status. Nevertheless, the χ^2^ analyses are robust to these unequal group sizes as the calculation of expected values takes the sample sizes into account.

### Conclusions

In summary, our study showed that a relatively small percentage of recreational endurance athletes across a variety of sports uses caffeine supplements to improve performance. Those who supplement with caffeine for training and races appear to base their supplementation strategies on self-experimentation and typical serving sizes of popular supplements, rather than on findings presented in the scientific literature. They typically take lower amounts of caffeine closer to the start of races and/or training sessions than what is commonly used in laboratory studies. However, the amounts are consistent with some scientific research investigating the effects of low-dose caffeine supplementation on endurance performance. The most popular mode of caffeine administration appears to be the ingestion of sports/energy gels, which also contain carbohydrate. While some studies have shown efficacy of these supplements compared to non-caffeinated placebos, it is unclear if they are as effective as pure caffeine in capsule or powder form. Additionally, the gels reported to be used by our participants contain less caffeine than those investigated in laboratory research.

Therefore, it appears that better education of recreational endurance athletes about amounts and timing of caffeine supplementation would be beneficial. While self-experimentation is a valid and effective way to establish nutrition and supplementation strategies, recreational endurance athletes might not have the appropriate knowledge to compare their self-selected strategies to more established research-based strategies. Further research using self-reported protocols would be useful to elucidate whether these strategies are successful when compared with placebo controls and more established strategies.

## Data Availability Statement

The datasets presented in this study can be found in online repositories. The names of the repository/repositories and accession number(s) can be found below: https://osf.io/9grc2/.

## Ethics Statement

The studies involving human participants were reviewed and approved by Texas Christian University Institutional Review Board. The patients/participants provided their written informed consent to participate in this study.

## Author Contributions

AK, AG, KM, RB-T, and MS: contributed to conception and design, acquisition of data, drafted and/or revised the article, and approved the submitted version for publication. AK and MS: contributed to analysis and interpretation of data. All authors contributed to the article and approved the submitted version.

## Funding

This work was supported in part by the Harris College of Nursing and Health Sciences Graduate Student Research Grant.

## Conflict of Interest

The authors declare that the research was conducted in the absence of any commercial or financial relationships that could be construed as a potential conflict of interest.

## Publisher's Note

All claims expressed in this article are solely those of the authors and do not necessarily represent those of their affiliated organizations, or those of the publisher, the editors and the reviewers. Any product that may be evaluated in this article, or claim that may be made by its manufacturer, is not guaranteed or endorsed by the publisher.

## References

[B1] Aguilar-Navarro M. Baltazar-Martins G. Brito de Souza D. Muñoz-Guerra J. Del Mar Plata M. Del Coso J. (2020). Gender differences in prevalence and patterns of dietary supplement use in elite athletes. Res. Q. Exerc. Sport. 20, 1–10. 10.1186/s12970-019-0296-5 32809924

[B2] Barzegar H. Arazi H. Mohebbi H. Sheykhlouvand M. Forbes S. C. (2021). Caffeine co-ingested with carbohydrate on performance recovery in national-level paddlers: a randomized, double-blind, crossover, placebo-controlled trial. J. Sports Med. Phys. Fit. 21, 5. 10.23736/S0022-4707.21.12125-5 34498818

[B3] Beaumont R. Cordery P. Funnell M. Mears S. James L. Watson P. (2017). Chronic ingestion of a low dose of caffeine induces tolerance to the performance benefits of caffeine. J. Sports Sci. 35, 1920–1927. 10.1080/02640414.2016.1241421 27762662

[B4] Bell D. G. McLellan T. M. (2002). Exercise endurance 1, 3, and 6 h after caffeine ingestion in caffeine users and nonusers. J. Appl. Physiol. 93, 1227–1234. 10.1152/japplphysiol.00187.2002 12235019

[B5] Booth F. W. Ruegsegger G. N. Toedebusch R. G. Yan Z. (2015). “Endurance Exercise and the Regulation of Skeletal Muscle Metabolism,” in Progress in Molecular Biology and Translational Science Molecular and Cellular Regulation of Adaptation to Exercise, ed. C. Bouchard (London: Academic Press), 129–151. 10.1016/bs.pmbts.2015.07.016 26477913

[B6] Chester N. Wojek N. (2008). Caffeine consumption amongst British athletes following changes to the 2004 WADA prohibited list. Int. J. Sports Med. 29, 524–528. 10.1055/s-2007-989231 18027309

[B7] Cooper R. Naclerio F. Allgrove J. Larumbe-Zabala E. (2014). Effects of a carbohydrate and caffeine gel on intermittent sprint performance in recreationally trained males. Eur. J. Sport Sci. 14, 353–361. 10.1080/17461391.2013.813972 23837918

[B8] Cox G. R. Desbrow B. Montgomery P. G. Anderson M. E. Bruce C. R. Macrides T. A.. (2002). Effect of different protocols of caffeine intake on metabolism and endurance performance. J. Appl. Physiol. 93, 990–999. 10.1152/japplphysiol.00249.2002 12183495

[B9] Davis J. M. Zhao Z. Stock H. S. Mehl K. A. Buggy J. Hand G. A. (2003). Central nervous system effects of caffeine and adenosine on fatigue. Am. J. Physiol. Regul. Integr. Comp. Physiol. 284, R399–404. 10.1152/ajpregu.00386.2002 12399249

[B10] de Morree H. M. Klein C. Marcora S. M. (2014). Cortical substrates of the effects of caffeine and time-on-task on perception of effort. J. Appl. Physiol. 117, 1514–1523. 10.1152/japplphysiol.00898.2013 25342703

[B11] de Salles Painelli V. Teixeira E. L. Tardone B. Moreno M. Morandini J. Larrain V. H.. (2021). Habitual caffeine consumption does not interfere with the acute caffeine supplementation effects on strength endurance and jumping performance in trained individuals. Int. J. Sport Nutr. Exerc. Metab. 31, 321–328. 10.1123/ijsnem.2020-0363 34010807

[B12] Del Coso J. Muñoz G. Muñoz-Guerra J. (2011). Prevalence of caffeine use in elite athletes following its removal from the World Anti-Doping Agency list of banned substances. Appl. Physiol. Nutri. Metabol. 36, 555–561. 10.1139/h11-052 21854160

[B13] Desbrow B. Barrett C. M. Minahan C. L. Grant G. D. Leveritt M. D. (2009). Caffeine, cycling performance, and exogenous CHO oxidation: a dose-response study. Med. Sci. Sports Exerc. 41, 1744–1751. 10.1249/MSS.0b013e3181a16cf7 19657295

[B14] Desbrow B. Leveritt M. (2006). Awareness and use of caffeine by athletes competing at the 2005 ironman triathlon world championships. Int. J. Sport Nutri. Exer. Metabol. 16, 545–558. 10.1123/ijsnem.16.5.545 17240785

[B15] Desbrow B. Leveritt M. (2007). Well-trained endurance athletes' knowledge, insight, and experience of caffeine use. Int. J. Sport Nutri. Exer. Metabol. 17, 328–339. 10.1123/ijsnem.17.4.328 17962708

[B16] Dodd S. L. Brooks E. Powers S. K. Tulley R. (1991). The effects of caffeine on graded exercise performance in caffeine naive versus habituated subjects. Eur. J. Appl. Physiol. Occup. Physiol. 62, 424–429. 10.1007/BF00626615 1893906

[B17] Doherty M. Smith P. M. (2005). Effects of caffeine ingestion on rating of perceived exertion during and after exercise: a meta-analysis. Scand. J. Med. Sci. Sport. 15, 69–78. 10.1111/j.1600-0838.2005.00445.x 15773860

[B18] Fralick A. M. Braun-Trocchio R. (2019). Division II athletes' dietary supplement use, sources of information, and motivations to use dietary supplements. J. Sport Behav. 41, 441−461.

[B19] Frary C. D. Johnson R. K. Wang M. Q. (2005). Food sources and intakes of caffeine in the diets of persons in the United States. J. Am. Diet Assoc. 105, 110–113. 10.1016/j.jada.2004.10.027 15635355

[B20] Froiland K. Koszewski W. Hingst J. Kopecky L. (2004). Nutritional supplement use among college athletes and their sources of information. Int. J. Sport Nutr. Exer. Metab. 14, 104–120. 10.1123/ijsnem.14.1.104 15129934

[B21] Gonçalves L. de S. Painelli V. de S. Yamaguchi G. Oliveira L. F. de Saunders B. da Silva R. P.. (2017). Dispelling the myth that habitual caffeine consumption influences the performance response to acute caffeine supplementation. J. Appl. Physiol. 123, 213–220. 10.1152/japplphysiol.00260.2017 28495846

[B22] Graham T. E. Spriet L. L. (1995). Metabolic, catecholamine, and exercise performance responses to various doses of caffeine. J. Appl. Physiol. 78, 867–874. 10.1152/jappl.1995.78.3.867 7775331

[B23] Grgic J. (2021). Effects of caffeine on resistance exercise: a review of recent research. Sports Med. 51, 2281–2298. 10.1007/s40279-021-01521-x 34291426

[B24] Grgic J. Grgic I. Pickering C. Schoenfeld B. J. Bishop D. J. Pedisic Z. (2019). Wake up and smell the coffee: Caffeine supplementation and exercise performance—an umbrella review of 21 published meta-analyses. Br. J. Sports Med. Bjspor. 018, 100278. 10.1136/bjsports-2018-100278 30926628

[B25] Grgic J. Mikulic P. (2021). Acute effects of caffeine supplementation on resistance exercise, jumping, and Wingate performance: no influence of habitual caffeine intake. Eur. J. Sport Sci. 21, 1165–1175. 10.1080/17461391.2020.1817155 32859145

[B26] Guest N. S. VanDusseldorp T. A. Nelson M. T. Grgic J. Schoenfeld B. J. Jenkins N. D. M.. (2021). International society of sports nutrition position stand: caffeine and exercise performance. J. Int. Soc. Sports Nutr. 18, 1. 10.1186/s12970-020-00383-4 33388079 PMC7777221

[B27] Harty P. S. Zabriskie H. A. Stecker R. A. Currier B. S. Tinsley G. M. Surowiec K.. (2020). Caffeine timing improves lower-body muscular performance: a randomized trial. Front. Nutr. 7, 585900. 10.3389/fnut.2020.585900 33330586 PMC7719671

[B28] Haytowitz D. B. Ahuja J. K. C. Wu X. Somanchi M. Nickle M. Nguyen Q. A.. (2019). USDA National Nutrient Database for Standard Reference, Legacy Release. USDA National Nutrient Database for Standard Reference, Legacy Release. Available online at: https://data.nal.usda.gov/dataset/usda-national-nutrient-database-standard-reference-legacy-release (accessed September 10, 2021).

[B29] Hogervorst E. Bandelow S. Schmitt J. Jentjens R. Oliveira M. Allgrove J.. (2008). Caffeine improves physical and cognitive performance during exhaustive exercise. Med. Sci. Sports Exer. 40, 1841–1851. 10.1249/MSS.0b013e31817bb8b7 18799996

[B30] Hulston C. J. Jeukendrup A. E. (2008). Substrate metabolism and exercise performance with caffeine and carbohydrate intake. Med. Sci. Sports Exerc. 40, 2096–2104. 10.1249/MSS.0b013e318182a9c7 18981939

[B31] Jagim A. R. Camic C. L. Harty P. S. (2019). Common habits, adverse events, and opinions regarding pre-workout supplement use among regular consumers. Nutrients 11, E855. 10.3390/nu11040855 31014016 PMC6520716

[B32] Jenkins N. T. Trilk J. L. Singhal A. O'Connor P. J. Cureton K. J. (2008). Ergogenic effects of low doses of caffeine on cycling performance. Int. J. Sport Nutri. Exer. Metabol. 18, 328–342. 10.1123/ijsnem.18.3.328 18562777

[B33] Jeukendrup A. E. (2004). Carbohydrate intake during exercise and performance. Nutrition 20, 669–677. 10.1016/j.nut.2004.04.017 15212750

[B34] Kamimori G. H. Karyekar C. S. Otterstetter R. Cox D. S. Balkin T. J. Belenky G. L.. (2002). The rate of absorption and relative bioavailability of caffeine administered in chewing gum versus capsules to normal healthy volunteers. Int. J. Pharm. 234, 159–167. 10.1016/S0378-5173(01)00958-9 11839447

[B35] Kim H.-Y. (2017). Statistical notes for clinical researchers: chi-squared test and Fisher's exact test. Restor. Dent. Endod. 42, 152–155. 10.5395/rde.2017.42.2.152 28503482 PMC5426219

[B36] Kovacs E. M. Stegen JHCH Brouns F. (1998). Effect of caffeinated drinks on substrate metabolism, caffeine excretion, and performance. J. Appl. Physiol. 85, 709–715. 10.1152/jappl.1998.85.2.709 9688750

[B37] Lane S. C. Hawley J. A. Desbrow B. Jones A. M. Blackwell J. R. Ross M. L.. (2014). Single and combined effects of beetroot juice and caffeine supplementation on cycling time trial performance. Appl. Physiol. Nutr. Metab. 39, 1050–1057. 10.1139/apnm-2013-0336 25154895

[B38] Lara B. Ruiz-Moreno C. Salinero J. J. Del Coso J. (2019). Time course of tolerance to the performance benefits of caffeine. PLoS One 14, e0210275. 10.1371/journal.pone.0210275 30673725 PMC6343867

[B39] Malito A. (2016). Think running is a cheap sport? Check out what New York City Marathon runners are spending. Available online at: https://www.marketwatch.com/story/the-new-york-city-marathon-is-this-weekend-guess-how-much-runners-spend-to-race-it-2016-11-04. (accessed October 5, 2021).

[B40] Mielgo-Ayuso J. Marques-Jiménez D. Refoyo I. Del Coso J. León-Guereño P. Calleja-González J. (2019). Effect of caffeine supplementation on sports performance based on differences between sexes: a systematic review. Nutrients 11, E2313. 10.3390/nu11102313 31574901 PMC6835847

[B41] Müller C. Jacobson K. A. (2011). Xanthines as adenosine receptor antagonists. Handb. Exp. Pharmacol. 10, 6. 10.1007/978-3-642-13443-2_6 20859796 PMC3882893

[B42] Paton C. Costa V. Guglielmo L. (2015). Effects of caffeine chewing gum on race performance and physiology in male and female cyclists. J. Sports Sci. 33, 1076–1083. 10.1080/02640414.2014.984752 25517202

[B43] Run Signup (2021). Annual Industry *Report* 2020.

[B44] Ryan E. J. Kim C.-H. Fickes E. J. Williamson M. Muller M. D. Barkley J. E.. (2013). Caffeine gum and cycling performance: a timing study. J. Strength Cond. Res. 27, 259–264. 10.1519/JSC.0b013e3182541d03 22476164

[B45] Scott A. T. O'Leary T. Walker S. Owen R. (2015). Improvement of 2000-m rowing performance with caffeinated carbohydrate-gel ingestion. Int. J. Sports Physiol. Perform. 10, 464–468. 10.1123/ijspp.2014-0210 25365032

[B46] Skinner T. L. Jenkins D. G. Folling J. Leveritt M. D. Coombes J. S. Taaffe D. R. (2013). Influence of carbohydrate on serum caffeine concentrations following caffeine ingestion. J. Sci. Med. Sport 16, 343–347. 10.1016/j.jsams.2012.08.004 22964452

[B47] Smirmaul B. P. C. de Moraes A. C. Angius L. Marcora S. M. (2017). Effects of caffeine on neuromuscular fatigue and performance during high-intensity cycling exercise in moderate hypoxia. Eur. J. Appl. Physiol. 117, 27–38. 10.1007/s00421-016-3496-6 27864638 PMC5306327

[B48] Southward K. Rutherfurd-Markwick K. J. Ali A. (2018). The effect of acute caffeine ingestion on endurance performance: a systematic review and meta-analysis. Sports Med. 48, 1913–1928. 10.1007/s40279-018-0939-8 29876876

[B49] Talanian J. L. Spriet L. L. (2016). Low and moderate doses of caffeine late in exercise improve performance in trained cyclists. Appl. Physiol. Nutri. Metabol. 41, 850–855. 10.1139/apnm-2016-0053 27426699

[B50] Vandivort M. (2019). State of the Sport: USAC Racing in 2019 by the Numbers. To Be Determined. Available online at: https://www.tobedetermined.cc/journal/state-of-the-sport-usac-racing-in-2019 (accessed November 22, 2020).

[B51] Venier S. Grgic J. Mikulic P. (2019). Caffeinated gel ingestion enhances jump performance, muscle strength, and power in trained men. Nutrients 11, E937. 10.3390/nu11040937 31027246 PMC6520843

[B52] Wiles J. D. Coleman D. Tegerdine M. Swaine I. L. (2006). The effects of caffeine ingestion on performance time, speed and power during a laboratory-based 1 km cycling time-trial. J. Sports Sci. 24, 1165–1171. 10.1080/02640410500457687 17035165

[B53] Yeo S. E. Jentjens R. L. P. G. Wallis G. A. Jeukendrup A. E. (2005). Caffeine increases exogenous carbohydrate oxidation during exercise. J. Appl. Physiol. 99, 844–850. 10.1152/japplphysiol.00170.2005 15831802

